# Murine Cytomegalovirus and Human Cytomegalovirus Differ in Pyroptosis Induction in Different Cell Types During Productive Replication

**DOI:** 10.3390/v17081106

**Published:** 2025-08-12

**Authors:** Jessica J. Carter, Daniel H. Schneider, Arshaan M. Hisamuddin, Richard D. Dix

**Affiliations:** 1 Viral Immunology Center, Department of Biology, Georgia State University, Atlanta, GA 30303, USA; jcarter80@gsu.edu (J.J.C.); dschneider6@student.edu.edu (D.H.S.); arshaanhisamuddin14@gmail.com (A.M.H.); 2 Department of Ophthalmology, Emory University School of Medicine, Atlanta, GA 30322, USA

**Keywords:** pyroptosis, murine cytomegalovirus, human cytomegalovirus, retinitis, MAIDS, AIDS, cell death suppression

## Abstract

Pyroptosis is a proinflammatory programmed cell death (PCD) that protects the host against invading viruses. We previously reported that pyroptosis plays a prominent role in the pathogenesis of murine cytomegalovirus (MCMV) retinal necrosis using mice with MAIDS as a mouse model for AIDS-related human cytomegalovirus (HCMV) retinal necrosis. Because MCMV and HCMV exhibit species specificity, we sought to determine if pyroptosis induction extends to different cell types of murine or human origin. In vitro studies were therefore performed in which MCMV-infected mouse fibroblasts and mouse macrophages were compared with HCMV-infected human fibroblasts and human ARPE-19 cells for stimulation of caspase-1, gasdermin G (GSDMD), and interleukin (IL)-18 and/or IL-1β transcripts as markers for canonical pyroptosis operation. Whereas MCMV stimulated significant stimulation of pyroptosis-associated transcripts during productive replication of mouse fibroblasts and mouse macrophages, significant stimulation of these transcripts was not detected during HCMV productive replication of human fibroblasts or ARPE-19 cells. Additional studies using UV-inactivated MCMV suggested that virion tegument proteins are not involved in the induction of pyroptosis in MCMV-infected mouse fibroblasts. We conclude that pyroptosis induction during productive replication of MCMV or HCMV is host cell type-dependent and may extend to species specificity, although virus-encoded PCD suppressors must be considered.

## 1. Introduction

Programmed cell death (PCD) has evolved as a network of signaling pathways intended to protect the host from invading pathogens, especially when resisting virus infection. Since the recognition of apoptosis over 50 years ago as a form of PCD [1], the past decade has witnessed the recognition of other forms of PCD that include necroptosis and pyroptosis (Table 1). Of these, pyroptosis is a caspase-dependent signaling pathway that serves as a critical mediator of host innate immune responses against invading viruses through the stimulation of a pronounced and sustained inflammation [2]. In response to pathogen-associated molecular patterns (PAMPs) through pattern recognition receptors (PRRs), activation of caspase-1-mediated pyroptosis (canonical pyroptosis) takes place via inflammasomes [3]. Inflammasomes typically are members of the leucine-rich repeat (LRR)-containing protein (NLR) family that includes NLRP1 and NLRP1b (a murine paralogue of NLRP1) as well as AIM2 that senses and binds to foreign cytoplasmic double-stranded DNA, including cytomegaloviruses. These multi-protein complexes form within the cytoplasm of the host cell, where pro-caspase-1 is cleaved to yield active caspase-1 that functions to further catalyze the proteolytic cleavage of gasdermin D (GSDMD). Subunits of GSDMD then localize to the plasma membrane where they form multiple pores resulting in osmotic lysis and cell death [4,5]. During cell lysis, however, there is also the processing and release of proinflammatory cytokines that include interleukin-18 (IL-18) and interleukin-1β (IL-1β) [6,7,8]. These proinflammatory cytokines conspire ultimately to induce the prominent inflammation associated with pyroptosis, thereby containing virus spread and minimizing virus-associated tissue destruction and clinical disease.

Pyroptosis has been suggested to operate during the pathogenesis of several human disease states, such as cancer [10], as well as during the evolution of several infectious diseases, such as AIDS [11] and COVID-19 [12]. Additional evidence has emerged pointing to a role for pyroptosis during the pathogenesis of several noninfectious retinal diseases that include age-related macular degeneration [13], retinitis pigmentosa [14], glaucoma [15], and retinal ischemia [16]. Missing conspicuously from this list of proptosis-associated retinal diseases, however, are those of virus origin, especially those caused by human herpesviruses [17]. For this reason, we have been pursuing investigations to define with some precision the relative contribution of pyroptosis toward the pathogenesis of human cytomegalovirus (HCMV) retinal necrosis, a sight-threatening retinal disease that develops in immunosuppressed persons, especially those with AIDS [18,19]. Toward this end, we have been performing studies using a well-characterized and clinically relevant mouse model of experimental murine cytomegalovirus (MCMV) retinal necrosis in mice with retrovirus-induced immunosuppression (MAIDS) to explore a possible role for pyroptosis during the onset and development of AIDS-related HCMV retinal necrosis. MAIDS is a progressive immunodeficiency of C57BL/6 mice induced by a mixture of mouse retroviruses that shares many immunopathologic features with AIDS pathogenesis in humans, including the appearance of chronic generalized lymphadenopathy, polyclonal B-cell activation, hypergammaglobulinemia, a Th1 to Th2 shift in cytokine production, and ultimate loss of CD4+ and CD8+ T-cell functions. Importantly, nearly 100% of MCMV-infected eyes of mice with MAIDS exhibit histopathologic features that mimic those found in the eyes of patients with AIDS-related HCMV retinal necrosis. These include the development of prominent cytomegalic cells contained within areas of full-thickness retinal necrosis that show complete destruction of the retinal architecture, as reviewed in [20,21]. Our findings to-date have revealed that MCMV-infected eyes of MAIDS mice deficient in one of three pyroptosis-associated inflammasomes (NLRP3, NLRP1b, or AIM2) [22] or deficient in one of four key components of the canonical pyroptosis pathway (caspase-1, GSDMD, IL-1β, or IL-18) [23] all exhibit similar if not identical patterns of atypical retinal pathology unlike the typical full-thickness retinal necrosis observed in MCMV-infected eyes of wildtype mice [24,25]. Thus, pyroptosis may play a significant role in the pathogenesis of MAIDS-related MCMV retinal necrosis.

All cytomegaloviruses are β-herpesviruses that exhibit species specificity. In addition to humans, cytomegaloviruses have been identified in several nonhuman primates, horses, guinea pigs, hamsters, rats, and mice [26]. Of these, MCMV often has been the cytomegalovirus of choice to investigate various aspects of the pathogenicity of HCMV due to striking similarities in their genomic structures, immunology, latency, and cellular/tissue tropisms [27,28]. We therefore extended our in vivo studies of pyroptosis and cytomegalovirus retinal necrosis in mice with MAIDS to a series of in vitro studies to compare and contrast MCMV and HCMV for their abilities to induce canonical pyroptosis during productive replication in different cell lines of physiologic importance. Specifically, mouse fibroblasts and mouse macrophages were infected with MCMV, whereas human fibroblasts and human retinal pigmented epithelial ARPE-19 cells were infected with HCMV. All were then assessed and compared quantitatively for stimulation of caspase-1, GSDMD, IL-18, and/or IL-1β transcripts at different times during productive virus replication for up to 72 to 120 h postinfection. Results revealed that MCMV-infected cells exhibited significant stimulation of pyroptosis-associated transcript markers early during productive replication. In sharp contrast, stimulation of pyroptosis-associated transcript markers by HCMV-infected cells during productive replication was relatively undetectable without significance at all times examined postinfection. These findings suggest that MCMV and HCMV differ in their induction of the canonical pyroptosis pathway during productive replication in a cell-type-specific manner that might also extend to species specificity.

## 2. Materials and Methods

### 2.1. Cell Lines

Mouse embryo fibroblasts (MEF) of C57BL/6 origin (No. SCRC-1002) obtained from American Type Culture Collection (ATCC) (Manassas, VA, USA) were grown in Dulbecco’s modified Eagle’s medium (DMEM) supplemented with 15% fetal bovine serum (FBS), 4 mM L-glutamine, 1% penicillin/streptomycin, 0.1 mg/mL gentamicin, and 1.5 g/L sodium bicarbonate and maintained according to ATCC recommendations.

IC-21 mouse macrophages of C57BL/6 origin (No. TIB-186) obtained from ATCC were grown in RPMI-1640 medium supplemented with 10% FBS, 1% penicillin/streptomycin, 0.1 mg/mL gentamicin, and 1.5 g/L sodium bicarbonate and maintained according to ATCC recommendations.

Human fetal lung fibroblasts (MRC-5) (No. CCL-171) obtained from ATCC were grown in DMEM supplemented with 10% FBS, 4 mM L-glutamine, 1% penicillin/streptomycin, 0.1 mg/mL gentamicin, and 1.5 g/L sodium bicarbonate and maintained according to ATCC recommendations.

Human retinal pigmented epithelial cells (ARPE-19) (No. CRL-2303) obtained from ATCC were grown in DMEM: F12 containing 10% FBS, 1% penicillin/streptomycin, 0.1 mg/mL gentamicin, and 1.5 g/L sodium bicarbonate and maintained according to ATCC recommendations.

### 2.2. Viruses

The Smith stain of MCMV was used throughout this investigation. Virus stocks were prepared via passage through salivary glands of adult female BALB/c mice (Harlan Laboratories, Greenfield, IN, USA) as previously described [24]. The use of BALB/c mice for preparation of MCMV virus stocks was approved by the Georgia State University Institutional Animal Care and Use Committee (IACUC).

The Towne strain of HCMV was used throughout this investigation. Virus stocks were prepared in monolayers of MRC-5 cells as described previously [29].

Quantification of MCMV and HCMV virus stocks was performed by standard plaque assay as described previously [24] using monolayers of MEF or monolayers of MRC-5 cells, respectively.

### 2.3. UV Inactivation of MCMV

UV inactivation of MCMV (UVi-MCMV) was accomplished by taking an aliquot of an infectious MCMV stock used in the same experiment and exposing it to DNA-damaging UV light [30]. This involved placing approximately 1 mL of infectious MCMV stock in an uncovered dish on ice at approximately 5 cm from the UV lamp source for three hours [31]. Complete inactivation of infectivity of the UV-MCMV stock was confirmed after incubation for 5 days of a monolayer of MEF inoculated with UV-MCMV without development of an infectious virus plaque.

### 2.4. Quantitative Real-Time RT-PCR Assay

Monolayers of MEF or IC-21 mouse macrophages were infected with MCMV (moi = 3), and monolayers of MRC-5 or ARPE-19 cells were infected with HCMV (moi = 3). Parallel mock-infected monolayers served as controls for all cell lines. At different times postinfection for each experiment (see figure legends), virus-infected and mock-infected cells were harvested by scraping into TRIzol^®^ reagent (Ambion/ThermoFisher Scientific, Waltham, MA, USA). Total RNA was then extracted with chloroform and purified over PureLink^®^ RNA Mini Kit spin cartridge filters (Ambion/ThermoFisher Scientific) according to the manufacturer’s instructions. RNA concentrations were determined using a Nanodrop 2000 spectrophotometer (Thermo Scientific, Pittsburgh, PA, USA) and normalized for each sample.

RNA for all samples was reverse-transcribed into cDNA using SuperScript™ III First-Strand Synthesis Kit reagents according to the manufacturer’s instructions (Invitrogen/ThermoFisher). Detection and quantification of target gene expression were performed via real-time RT-PCR assay using Applied Biosystems 7500 Fast Real-Time PCR System hardware and software in conjunction with Power SYBR Green Master Mix (Applied Biosystems, Foster City, CA, USA). All primers were obtained commercially from Qiagen (Valencia, CA, USA). These included mouse-specific primers for caspase-1 (QT00199458), GSDMD (QT00107247), IL-18 (QT00171129), IL-1β (QT01048355), and glyceraldehyde-3-phosphate dehydrogenase (GAPDH) (QT01192646), and human-specific primers for caspase-1 (QT00001568), GSDMD (QT01155448), IL-18 (QT00014560), IL-1β (QT00081263), and GAPDH (QT00079247). All samples were analyzed using the following thermocycling parameters: 10 min at 95 °C followed by 40 cycles consisting of 15 s at 94 °C, 31 s at 55 °C, and 35 s at 70 °C. Cycles to the threshold (CT) for each target gene were determined, and the ΔCT value for each sample was normalized by subtracting the CT value of its own endogenous housekeeping gene (GAPDH) from the CT value of the target gene. The ΔCT values of each target gene mRNA were compared with mock-infected controls for each time point by the 2^−ΔΔCt^ method to determine the change in gene expression, thereby yielding a relative fold change in mRNA expression for each group.

### 2.5. Statistical Analysis

Statistical analysis was performed using GraphPad Prism^®^ v8.2.1 software with a significance level (α) set to 0.05; *p*-values of <0.05 were considered statistically significant. Virus-infected samples were compared with mock-infected (control) samples collected at the same time points postinfection using a two-way analysis of variance (ANOVA). At least two independent experiments were performed. Data points represent mean fold changes ± standard deviations (SDs).

## 3. Results

### 3.1. Pyroptosis-Associated Transcripts Are Stimulated During Productive Replication of MCMV in Two Different Mouse Cell Types

When compared with other human herpesviruses such as herpes simplex virus type 1 (HSV1), the replication cycle for all cytomegaloviruses is relatively slow, and, importantly, cytomegalovirus replication does not immediately kill the host cell or shut off host cell metabolic processes as occurs with HSV1 [32]. For MCMV, 24 to 36 h is required to yield detectable levels of progeny virus following initial infection [33]. Like all cytomegaloviruses, this productive replication of MCMV takes place through a coordinated and temporal cascade regulation of virus-encoded protein synthesis that is divided sequentially into distinct kinetic classes that include the expression of immediate–early proteins, early/delayed proteins, and late protein products, and with virus DNA synthesis occurring 14 to 16 h postinfection [33]. The timing of pyroptosis induction relative to the course of productive replication of MCMV or any other virus probably occurs within hours after virus entry, although opposing virus-encoded mechanisms may also serve to delay or abolish pyroptosis induction to enhance replication [9,34]. The timing of pyroptosis appearance may also be modulated by host cell type due to differences in activation of inflammasomes in response to PAMPs through PRRs that may vary from cell type to cell type [35]. With this background information, we compared two different mouse cell types for the induction of canonical pyroptosis-associated transcripts at different times after MCMV infection.

**Figure 1:**
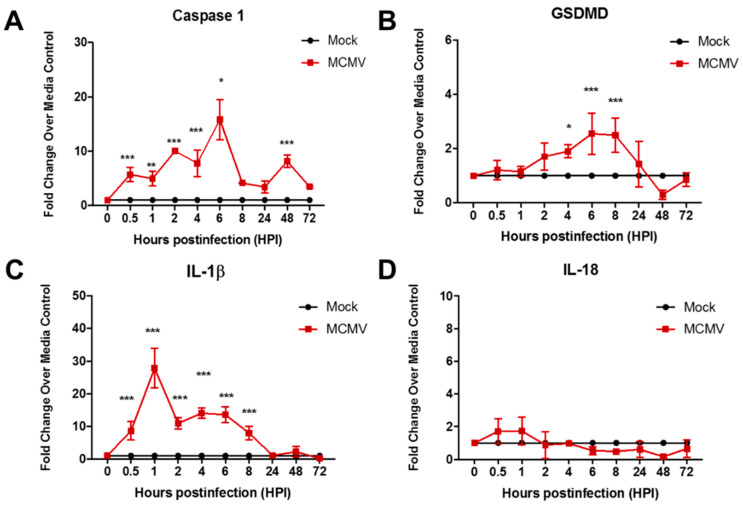
Monolayers of MEF were infected with MCMV (moi = 3 PFU/cell) or mock-infected with cell culture maintenance medium (control). At 0.5, 1, 2, 4, 6, 8, 24, 48, and 72 h postinfection, monolayers were collected and assessed by real-time RT-PCR assay for detection and quantification of (**A**) caspase-1 mRNA, (**B**) GSDMD mRNA, (**C**) IL-1β mRNA, and (**D**) IL-18 mRNA. Means ± SD of duplicate experiments are shown. * *p* < 0.5; ** *p* < 0.01; *** *p* < 0.001 (ANOVA).

Initial experiments were performed using the mouse embryo fibroblast (MEF) cell line because, historically, fibroblasts have been the cell type of choice for investigations of permissive MCMV replication [36]. MEF monolayers were infected with MCMV, collected at various times postinfection, and analyzed for detection and quantification of caspase-1, GSDMD, IL-1β, and IL-18 transcripts, all as markers for the operation of pyroptosis by real-time RT-PCR assay. Parallel mock-infected MEF monolayers served as controls for all time points examined. The results are shown in Figure 1.

When compared with mock-infected MEF controls, MCMV-infected MEF exhibited significant and robust stimulation of caspase-1, GSDMD, and IL-1β transcript production at 0.5 to 8 h postinfection and therefore early during the course of productive MCMV replication. Surprisingly, stimulation of pyroptosis-associated IL-18 transcript was relatively undetectable and without statistical significance.

**Figure 2:**
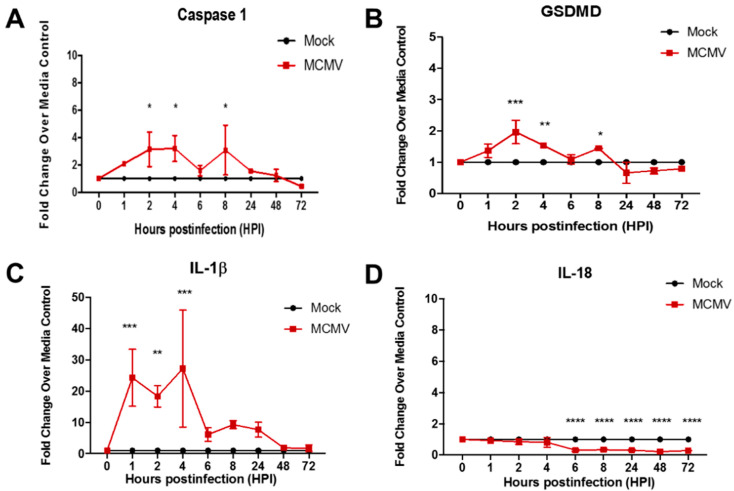
Monolayers of IC-21 mouse macrophages were infected with MCMV (moi = 3 PFU/cell) or mock-infected with cell culture maintenance medium (control). At 1, 2, 4, 6, 8, 24, 48, and 72 h postinfection, monolayers were collected and assessed by real-time RT-PCR assay for detection and quantification of (**A**) caspase-1 mRNA, (**B**) GSDMD mRNA, (**C**) IL-1β mRNA, and (**D**) IL-18 mRNA. Means ± SD of duplicate experiments are shown. * *p* < 0.5; ** *p* < 0.01; *** *p* < 0.001; **** no statistical significance (ANOVA).

Because macrophages are involved in the pathogenesis of AIDS-related HCMV retinal necrosis [37] as well as MAIDS-related MCMV retinal necrosis [38], parallel studies were next performed using the IC-21 mouse macrophage cell line to compare the temporal patterns of MCMV-induced caspase-1, GSDMD, IL-1β, and IL-18 transcript production for this mouse cell type with those obtained for MCMV-infected MEF. It is noteworthy that MCMV-infected IC-21 mouse macrophages were used by us previously to track virus from peripheral blood to retinal tissues in mice with MAIDS [39]. The results are shown in Figure 2. In agreement with findings obtained for MCMV-infected MEF, MCMV-infected IC-21 mouse macrophages displayed significant stimulation of caspase-1, GSDMD, and IL-1β transcripts early during the course of virus replication (2–8 h postinfection), although not as robust as that seen in MCMV-infected MEF. Once again, however, production of the IL-18 transcript was undetectable at all times postinfection examined and was similar to that observed for MCMV-infected MEF.

Taken together, the detection of significant stimulation of transcripts for two key components of the pyroptosis pathway, caspase-1 and especially GSDMD, as well as the pyroptosis-associated proinflammatory cytokine IL-1β early during the course of MCMV productive replication, suggests that the canonical pyroptosis pathway is induced and operates during MCMV infection of two different mouse cell lines. The intensity of pyroptosis induction, however, appears to differ between mouse fibroblasts and mouse macrophages, suggesting that the degree of pyroptosis induction by MCMV may be host cell-type specific.

### 3.2. Pyroptosis-Associated Transcripts Are Not Stimulated During Productive Replication of HCMV in Two Different Mouse Cell Types

The kinetics of productive replication of HCMV are similar, but not identical, to those of MCMV, with HCMV showing a slower replication cycle. Although immediate–early gene products are expressed rapidly following HCMV infection, early/delayed and late gene expression do not occur until 24 to 36 h postinfection, and with HCMV DNA replication not detected until 14 to 16 h postinfection [33]. Infectious progeny virus particles begin to accumulate after 48 h and reach maximum levels at 72 to 96 h postinfection. In the clinical setting, it is estimated that the doubling time for HCMV replication in the blood is approximately 1 day [40,41]. With this information on the dynamics of HCMV replication in mind, we performed an additional set of studies to explore the possible induction of pyroptosis during productive replication of HCMV in two different human cell lines.

**Figure 3:**
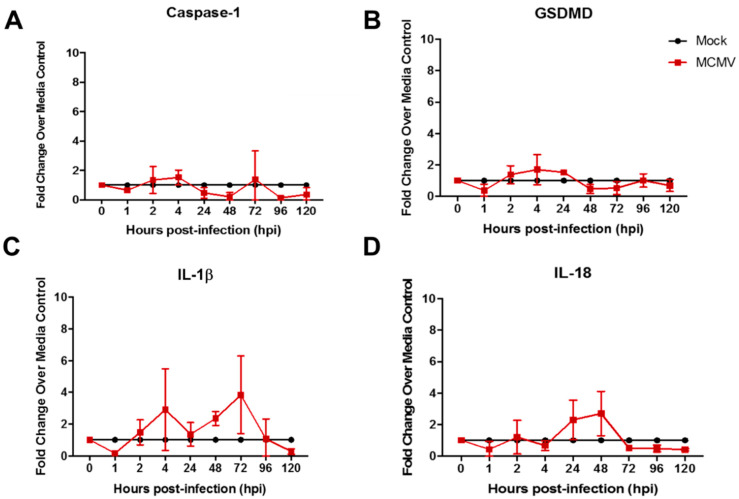
Monolayers of MRC-5 human fibroblasts were infected with HCMV (moi = 3 PFU/cell) or mock-infected with cell culture maintenance medium (control). At 1, 2, 4, 24, 48, 72, 96, and 120 hr postinfection, monolayers were collected and assessed by real-time RT-PCR assay for detection and quantification of (**A**) caspase-1 mRNA, (**B**) GSDMD mRNA, (**C**) IL-1β mRNA, and (**D**) IL-18 mRNA. Means ± SD of duplicate experiments are shown. No statistical significance was observed for any transcript at all times examined. Key applies to all panels.

The first HCMV studies were performed using the MRC-5 human fibroblast cell line because fibroblasts are permissive for HCMV replication and clinically significant [33]. MRC-5 monolayers were infected with HCMV, collected at various times postinfection (but at times exceeding those for MCMV studies due to the slower replication cycle of HCMV), and analyzed for detection and quantification of caspase-1, GSDMD, and IL-18 and/or IL-1β transcripts by real-time RT-PCR assay as for prior MCMV studies. Parallel mock-infected monolayers served as controls for all time points examined. In sharp contrast to MCMV-infected MEF (a mouse fibroblast cell line) (Figure 1), HCMV-infected MRC-5 (a human fibroblast cell line) failed to show significant stimulation for any pyroptosis-associated transcripts at all times examined postinfection, including those for caspase-1, GSDMD, IL-1β, and IL-18 (Figure 3).

**Figure 4:**
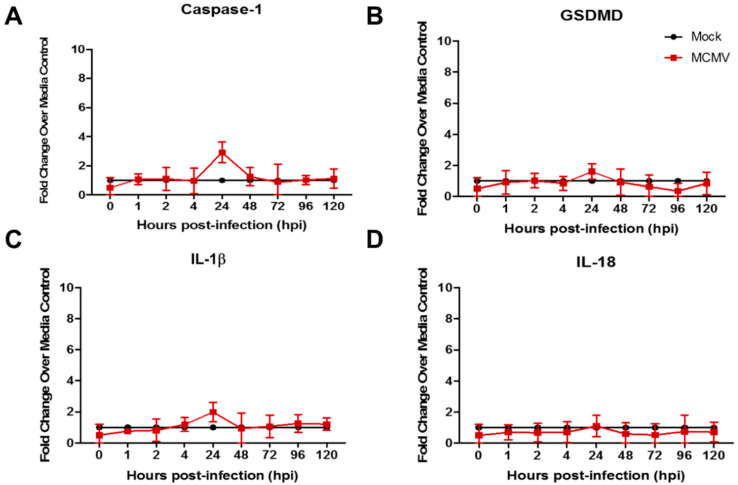
Monolayers of human ARPE-19 cells fibroblasts were infected with HCMV (moi = 3 PFU/cell) or mock-infected with cell culture maintenance medium (control). At 1, 2, 4, 24, 48, 72, 96, and 120 hr postinfection, monolayers were collected and assessed by real-time RT-PCR assay for detection and quantification of (**A**) caspase-1 mRNA, (**B**) GSDMD mRNA, (**C**) IL-1β mRNA, and (**D**) IL-18 mRNA. Means ± SD of duplicate experiments are shown. No statistical significance was observed for any transcript at all times examined. Key applies to all panels.

To provide further clinical relevance to our ongoing investigations of cytomegalovirus retinal necrosis pathogenesis, we next performed a parallel study using the ARPE-19 human cell line because retinal pigmented epithelium (RPE) undergo productive cytomegalovirus replication in mice with MAIDS-related MCMV retinal necrosis and in patients with AIDS-related HCMV retinal necrosis as evidenced by the appearance of diagnostic cytomegalic inclusion bodies in both mice and humans [24,42]. Similar to results using a human fibroblast cell line but in opposition to results obtained for MCMV-infected mouse fibroblast or mouse macrophage cell lines (Figure 1 and Figure 2), HCMV-infected ARPE-19 cells also failed to demonstrate significant stimulation of transcripts for caspase-1, GSDMD, IL-1β, or IL-18 at all times examined postinfection (Figure 4).

Thus, whereas two different mouse cell lines upon MCMV infection demonstrated evidence for the induction and operation of the canonical pyroptosis pathway, two different human cell lines upon HCMV infection failed to induce significant amounts of any of the canonical pyroptosis pathway-associated transcripts investigated throughout the course of HCMV productive replication.

### 3.3. Pyroptosis-Associated Transcripts Are Not Stimulated in Mouse Fibroblasts Following Inoculation with UV-Inactivated MCMV

**Figure 5:**
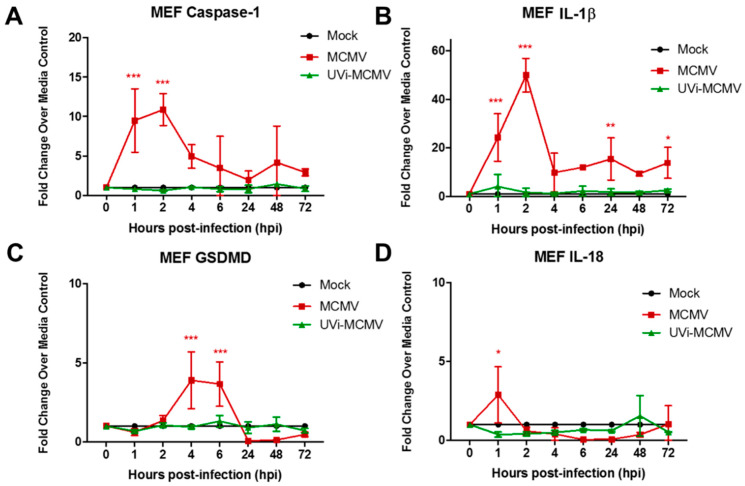
Monolayers of MEF were inoculated with infectious MCMV (moi = 3 PFU/cell), an equal volume of UV-inactivated MCMV (UVi-MCMV), or mock-infected with cell culture maintenance medium (control). At 1, 2, 4, 24, 48, and 72 h postinfection, monolayers were collected and assessed by real-time RT-PCR assay for detection and quantification of (**A**) caspase-1 mRNA, (**B**) IL-1β mRNA, (**C**) GSDMD mRNA, and (**D**) IL-18 mRNA. Means ± SD of duplicate experiments are shown. * *p* < 0.5; ** *p* < 0.01; *** *p* < 0.001 (ANOVA).

Exposure of MCMV to DNA-damaging UV light renders the virus deficient in virus gene expression and blocks subsequent productive replication but allows attachment, adsorption, and release of tegument proteins into the host cell. We have used this experimental approach previously to determine if tegument proteins are sufficient for suppressor of cytokine signaling (SOCS)1 and SOCS3 transcript stimulation during MCMV infection of MEF or IC-21 mouse macrophages [43]. Accordingly, we inoculated monolayers of MEF with either infectious MCMV or UV-inactivated MCMV (UV-MCMV), collected each at identical times postinfection, and analyzed and compared each for detection and quantification of caspase-1, GSDMD, IL-18, and/or IL-1β transcripts by real-time RT-PCR assay. As expected, and in agreement with earlier studies (Figure 1), MEF inoculated with infectious MCMV when compared with mock-infected MEF once again exhibited significant and robust stimulation of caspase-1, GSDMD, and IL-1β transcripts, but also IL-18 transcript production, at 1 to 4 or 6 h postinfection. In sharp contrast, MEF inoculated with UVi-MCMV failed to display significant stimulation for any pyroptosis-associated transcript for all times examined postinfection, a finding also observed for mock-infected MEF (Figure 5).

These results collectively suggest that the release of virus-encoded tegument proteins into the host cell plays no role in the induction of the canonical pyroptosis pathway during MCMV productive replication of mouse fibroblasts.

## 4. Discussion

The three most studied cell death programs that operate during an innate immune response to virus infection are apoptosis, necroptosis, and pyroptosis (Table 1). Of these, we have focused our attention most recently on pyroptosis. Unlike apoptosis and necroptosis, caspase-1-mediated pyroptosis is activated by inflammasomes that ultimately promote the processing and release of proinflammatory cytokines, including IL-18 and IL-1β [6,7,8] that orchestrate the intense inflammatory phenotype that is the hallmark feature of pyroptosis [2]. Because we have been using MAIDS-related MCMV retinal necrosis as a mouse model to investigate the pathogenesis of AIDS-related HCMV retinal necrosis [20,21], we have become interested in possible host cell type-specific as well as possible species-specific differences when comparing MCMV and HCMV for pyroptosis induction. Indeed, different cell types can assemble distinct inflammasome complexes that could lead to diverse immunologic outcomes [35], especially during virus infection. This concept therefore becomes especially important when considering the great diversity of cell types that comprise the mouse and human retinas as possible targets for MCMV and HCMV infection and replication during retinal disease pathogenesis.

Herein, we provide new evidence that MCMV and HCMV differ remarkably in their comparative abilities to induce key pyroptosis-associated transcripts during productive replication in mouse cell lines or human cell lines, respectively. Two different mouse cell lines resulted in detectable and significant stimulation of caspase-1, GSDMD, and IL-1β transcripts in response to MCMV infection within hours after initial inoculation. Surprisingly, pyroptosis induction by mouse fibroblasts was far more vigorous when compared with pyroptosis induction by mouse macrophages, an observation suggesting possible cell-type-dependent differences in the intensity of pyroptosis stimulation. Unlike MCMV, however, parallel in vitro studies pursued during HCMV productive replication in two different human cell lines showed no significant stimulation for any key pyroptosis-associated transcripts examined for up to 120 h postinfection. From a host cell perspective, one possible interpretation of these results is that the intensity of pyroptosis induction by MCMV or HCMV infection is cell-type specific, which may also extend to species specificity.

It is also important to interpret our findings from a virus perspective, however. Cytomegaloviruses, including MCMV and HCMV, successfully deploy virus-induced suppressors to counteract host cell death signaling pathways [9,34]. This mechanism allows these viruses to prolong productive replication and release of progeny virus to increase the duration and range of acute infection. For example, MCMV and HCMV retain homologues capable of suppressing caspase-8-mediated apoptosis across species lines [44]. Table 1 summarizes suppression of extrinsic apoptosis, necroptosis, and canonical pyroptosis by specific MCMV or HCMV-encoded genes. Thus, our inability to detect significant amounts of key pyroptosis-associated transcripts during HCMV productive replication in both human MRC-5 and ARPE-19 cell lines may be due to expression of the HCMV UL83 gene (and possibly the UL84 gene) that has been shown to impact AIM2 inflammasome sensing and signaling [9,45] as a mechanism to dampen or block canonical pyroptosis. An alternative, but unlikely, possibility is that neither MRC-5 nor ARPE-19 cell lines possess the host cell machinery to induce pyroptosis and, therefore, are unable to stimulate any of the pyroptosis-associated transcripts under investigation. Whether other PCD pathways operate in these cell lines in response to HCMV infection remains unknown.

When compared with HCMV, MCMV M84 gene encodes for an amino acid homologue to the UL83 gene product that also suppresses AIM2 inflammasome function by interacting with the pyrin domain-containing adaptors of AIM2, thereby counteracting inflammasome activation and caspase-1-mediated pyroptosis [46]. Poor replication of M84-deficient MCMV in macrophages was reported by the Brune laboratory due to increased production of proinflammatory cytokines and pyroptosis [34,46]. Nonetheless, we observed detectable stimulation of key pyroptosis-associated transcripts in an MCMV-infected macrophage cell line and even more robust stimulation of these transcripts in MCMV-infected fibroblasts, even though these could not be detected significantly in HCMV-infected fibroblasts. It is therefore unclear as to why MCMV-encoded M84 should not operate to dampen or block pyroptosis induction in these two mouse cell lines, especially in mouse macrophages as reported previously [46]. Two possibilities present themselves to explain these findings. Firstly, MCMV strains may differ in their abilities to induce pyroptosis. We can dismiss this possibility when comparing our study with that of Deng and coworkers [46] because both used the Smith strain of MCMV. Nonetheless, we are now comparing the Smith and K181 strains of MCMV for their pyroptosis induction potentials in vitro and in vivo. Secondly, although mouse macrophages were used in both studies, we employed IC-21 mouse macrophages, whereas Deng and coworkers [46] performed their investigations using J774A.1 mouse macrophages and immortalized bone marrow-derived macrophages. Whether different mouse macrophage cell lines exhibit cell-line-distinct pyroptosis induction outcomes during the course of MCMV productive replication remains to be determined.

Herein, we also provide new evidence that the induction of canonical pyroptosis in the MEF cell line during MCMV productive replication does not involve tegument proteins that are released into the host cell immediately after virus attachment and adsorption of the parental virion. Tegument proteins of cytomegaloviruses are structural proteins (of which many are phosphoproteins) [47] that mediate several key events during productive replication, including efficient assembly of infectious progeny virus and virus genome replication, but also play a role in the activation of virus-encoded immediate–early gene expression necessary for the initiation of productive virus replication [48]. If one or more MCMV-encoded tegument proteins play a role in pyroptosis induction, stimulation of pyroptosis-associated transcripts should be readily detectable in the mouse fibroblast cell line after inoculation with UV-inactivated parental virus particles that are deficient in the expression of all kinetic classes of virus gene expression due to DNA-damaging UV light [30]. This was not the case. When compared with MEF inoculated with infectious MCMV that showed robust stimulation of caspase-1, GSDMD, and IL-1β transcripts at early times postinfection, MEF inoculated with UV-inactivated MCMV failed to show significantly detectable transcripts at all times postinfection for any of the components of the canonical pyroptosis pathway examined. Importantly, MEF inoculated with UV-inactivated MCMV or mock-infected demonstrated identical experimental outcomes. This observation is consistent with the MCMV-induced M84 gene being involved in pyroptosis induction. The M84 gene product is not a tegument protein, but a nonstructural protein that is expressed as an early/delayed protein during productive virus replication [49,50]. Its expression would therefore be prevented by the UV-inactivation procedure that would abolish expression of all kinetic classes of virus-induced protein synthesis, including all early/delayed protein expression.

## 5. Conclusions

We provide new evidence that induction of the canonical pyroptosis pathway during the productive replication of MCMV or HCMV in different host cell types differs significantly and may be species-specific. The results are summarized in Table 2. Whereas MCMV stimulated the significant production of several pyroptosis-associated transcripts that included those for caspase-1, GSDMD, and IL-1β during productive replication in mouse fibroblast and mouse macrophage cell lines, significant stimulation of these transcripts was not detected during HCMV productive replication in human fibroblast and ARPE-19 cell lines. These findings must be viewed, however, through the “lens of cell death suppression” as reviewed by Mocarski [9] that is induced by the M84 gene of MCMV and the UL83 gene of HCMV. Additional studies revealed that pyroptosis induction in a MCMV-infected mouse fibroblast cell line did not involve one or more structural tegument proteins of the parental virus following initial attachment and adsorption events. Taken together, these in vitro findings complement our previous in vivo investigations that have demonstrated a significant contribution of inflammasomes and the canonical pyroptosis pathway toward the onset and development of full-thickness retinal necrosis in MCMV-infected eyes of mice with MAIDS [22,23] and further place these findings in the context of AIDS-related retinal necrosis pathogenesis induced by HCMV.

## Figures and Tables

**Table 1:** Comparison of apoptosis, pyroptosis, and necroptosis cell death signaling pathways relative to MCMV and HCMV infection.

	Signaling Pathway	Consequences	Proinflammatory?	Pathway Suppression via Virus-Encoded Gene ^a^
**Extrinsic Apoptosis**	TNFR1 TNF Caspase-8 Caspase-3	Cell lysis Phagocytosis	No	M36 (MCMV) UL36 (HCMV)
**Canonical Pyroptosis**	Inflammasomes Caspase-1 Gasdermin D	Cell lysis Release of IL-1β and IL-18	Yes	M84 (MCMV) UL83 (HCMV)
**Necroptosis**	RIPK1 RIPK3 MLKL	Cell lysis Release of cellular contents	Yes	M45 (MCMV)

^a^ Summarized by Mocarski [9].

**Table 2:** Summary of results for pyroptosis induction by MCMV or HCMV in difference cell types.

	UV-inactivated MCMV	MCMV	HCMV
**Cell Type**	Mouse fibroblasts Mouse macrophages	Mouse fibroblasts Mouse macrophages	Human fibroblasts ARPE-19 cells
**Evidence for** **Pyroptosis Induction**	No	Yes	No
		Stimulation of caspase-1 GSDMD, and IL-1β transcripts	

## Data Availability

The original contributions presented in this study are included in this article. Further inquiries can be directed to the corresponding author.
